# Mistletoe as an Oxytoxic

**Published:** 1878-06

**Authors:** 


Mistletoe as an Oxytoxic.—About three years ago I was informed by my friend, Dr. W. H. Long, of Louisville, of the superior properties of viscum album (mistletoe) as an oxytoxic. I then determined at the first opportunity to try its merits. During last fall I had some leaves of the viscum album gathered, and I made an infusion. Not knowing the exact proportions, I made it according to the general rule of infusions, two ounces to the pint; but not waiting for the leaves to dry, I used them green, and doubled the quantity.

*Case* 1.—November, 1877, Mrs. McC., mult., had advanced to the second stage of labor, and there remained for several hours. Upon close examination I concluded that all that was wanting was an efficient action of the uterus. Having an eight-ounce bottle of the infusion in my pocket, I gave about one-third of it, and in twenty minutes I gave her another third; and in ten minutes more regular clonic contractions of the uterus began, and during the hour she was safely delivered of both infant and placenta.

*Case* 2.—December, 1877. Mrs. P., mult., advanced to second stage of labor, according to the "granny's" statement, from noon till after dark, when I was sent for. After learning the facts in the case, I gave Mrs. P. four ounces of the infusion, and very efficient uterine contractions followed in twenty minutes. The pains recurred about every three or four minutes; yet, after an hour, seemingly no impression had been made upon the child's head. Upon closer examination I found an occipito-posterior position which had missed the usual rotation of the occiput under the pubic arch. The occipito-mental diameter corresponded with the anterio-posterior diameter of the cavity. The head seemed to be locked in this position, which prevented flexion, notwithstanding the continued and powerful contractions of the uterus. I introduced a pair of perineal forceps, and with a slight traction and flexion dislodged the head from what seemed to be its locked position. The next pain was so powerful that the child and secundines were delivered at once. So, notwithstanding the delay after the dose of viscum, we see that it was not for want of action of the drug on the uterus.

*Case* 3.—January, 1878, Mrs. C., having suffered from menorrhagia for eight months, applied to me for relief. She stated that her menses recurred about every eighteen or twenty days, and that the discharge was very profuse during the first two days; said she was greatly weakened from the loss of so much blood; looked pale. I gave her one-ounce doses of the infusion, to be taken just before and during the next recurrence, and to continue it when her menses recurred out of time or too profusely. She reported herself a few weeks since as regular, and feeling much improved.

My short experience with the parasite is, that it acts more promptly and more decidedly as an oxytoxic than ergot. In the few cases that I have used it I have had none of the troublesome "after-pains" that are often observed when ergot has been given.

In administering ergot I never know whether to look for its oxytoxic effects or not, it is so uncertain; yet this may be the fault of the manufacturer. Be that as it may, I, for one, will herald the introduction of the new oxytoxic (viscum album) into our materia medica with much satisfaction, giving all due honors to its introducer, our friend, Dr. W. H. Long.—*Louisville Medical News.*

